# Large sub-clonal variation in *Phytophthora infestans* from recent severe late blight epidemics in India

**DOI:** 10.1038/s41598-018-22192-1

**Published:** 2018-03-13

**Authors:** Tanmoy Dey, Amanda Saville, Kevin Myers, Susanta Tewari, David E. L. Cooke, Sucheta Tripathy, William E. Fry, Jean B. Ristaino, Sanjoy Guha Roy

**Affiliations:** 1 Department of Botany, West Bengal State University, Kolkata, 700126 India; 2 Department of Entomology and Plant Pathology, North Carolina State University, Raleigh, North Carolina, Raleigh, NC 27607 USA; 3 Plant Pathology and Plant-Microbe Biology, Cornell University, Ithaca, NY14850 USA; 4 TeraBundle Anlytics Pvt. Ltd, Haldia, 721602 India; 5 James Hutton Institute, Dundee, DD2 5DA UK; 6 Indian Institute of Chemical Biology, Kolkata, 700032 India; 7 Academy of Scientific and Innovative Research, Ghaziabad, Delhi, India

## Abstract

The population structure of the *Phytophthora infestans* populations that caused the recent 2013–14 late blight epidemic in eastern India (EI) and northeastern India (NEI) was examined. The data provide new baseline information for populations of *P*. *infestans* in India. A migrant European 13_A2 genotype was responsible for the 2013–14 epidemic, replacing the existing populations. Mutations have generated substantial sub-clonal variation with 24 multi-locus genotypes (MLGs) found, of which 19 were unique variants not yet reported elsewhere globally. Samples from West Bengal were the most diverse and grouped alongside MLGs found in Europe, the UK and from neighbouring Bangladesh but were not linked directly to most samples from south India. The pathogen population was broadly more aggressive on potato than on tomato and resistant to the fungicide metalaxyl. Pathogen population diversity was higher in regions around the international borders with Bangladesh and Nepal. Overall, the multiple shared MLGs suggested genetic contributions from UK and Europe in addition to a sub-structure based on the geographical location within India. Our data indicate the need for improved phytosanitary procedures and continuous surveillance to prevent the further introduction of aggressive lineages of *P*. *infestans* into the country.

## Introduction

The late blight pathogen *Phytophthora infestans* (Mont.) de Bary has had a major impact on both science^1,2^ and society^3,4^ and continues to do so even 170 years after the first reported outbreaks. Its aggressiveness, co-evolution with its host and a high mutation rate make *P*. *infestans* the most important among oomycete pathogens^1^, and a persistent threat to potato production. Potatoes are the third most important staple crop behind wheat and rice, and the pathogen represents a significant threat to food security^5,6^. Compared to other stramenopiles, *P*. *infestans* has a large genome (240 Mb) with high levels of repeat rich sequences^7^. The origin of this ‘economically important pathogen’^4^ has been debated with work suggesting either an Andean origin in South America^2,8–10^ or a central Mexican^11–14^ origin. The pathogen caused widespread disease first in the US and then migrated to Europe and other parts of the world and has historically been disseminated as a series of clonal lineages. The lineage containing the HERB-1 mtDNA haplotype and the FAM-1 nuclear genotype initiated the Irish famine in 1840s^9,10,15,16^ and was dominant and widespread in various parts of the world during the 19^th^ century^16^. This lineage was later replaced by the US-1 genotype^15–17^ which dominated until the 1980s^18^ and was, in turn, displaced by other aggressive clonal lineages worldwide^19,20^.

The population structure of *P*. *infestans* has continued this rapid change over the past 40 years but variation in its reproductive biology and thus its genetic diversity is observed globally. The pathogen is heterothallic with two mating types, A1 and A2, and the presence of both types in central Mexico and in the Nordic countries of Europe and the Netherlands has led to sexual reproduction and high genetic diversity^13,21,22^. In Western Europe however, the *P*. *infestans* population is dominated by aggressive clonal genotypes including 13_A2 (also known as Blue_13) and 6_A1^23,24^. The highly aggressive and dominant European genotype 13_A2 was first recorded in 2004 in the Netherlands and Germany^23^ and has subsequently been found in China^25^ and India^26^. The spread of new aggressive lineages has led to an increase in late blight incidence in India and represents a current threat to food security^27^. Many other late blight epidemics were also reported during 1990 to 2014 throughout the world. Among these outbreaks, the most recent and severe one in India occurred in the eastern and north-eastern regions, primarily in the state of West Bengal in 2014^28^.

India is the 2^nd^ largest producer of potato with 80% of the total production in the northern and eastern parts of the country. Potato is a winter crop in most parts of India. Exceptions are in the northern and north-eastern hills, where potato is a summer crop, and the southern regions, where potato is an autumn crop. Late blight is a recurring problem in the northern hills every year. In the Indo-Gangetic plains, where the major growing regions of India are located, the disease is mild to sporadic each year. However, once every two to three years, the pathogen becomes epiphytotic, causing up to 75% loss^29^. In the plains of eastern India, crop rotation is routine with potato grown between November and February in fields that are used for rice cultivation for the rest of the year. These host dynamics affect the pathogen population through repeated extinction and re-colonization events and this may affect gene flow as well as evolutionary trajectories. There are records of at least four migrations of *P*. *infestans* into India over the past 100 years. For example, the oldest samples of *P*. *infestans* from 1913 collected by J. F. Dastur in Bhagalphur (Bihar) were the Ia mitochondrial haplotype^30^ and the US-1 clonal lineage (Ib haplotype) was present in India by the 1960s^30^. The presence of the A2 mating type in 1990s in northern hills provided additional evidence suggesting migration from an outside source^31^ and more recently, the European 13_A2 genotype was detected in southern India^26^ and dominant on both potato and tomato between 2010 and 2012^32^. However, no clear population structures have been characterized in subtropical Indo-Gangetic regions and northeastern parts of India, which share many international borders with Nepal and Bangladesh. West Bengal, Assam, and Meghalaya are adjacent to Bangladesh where late blight is reported on potatoes cultivated under similar cropping regimes and climate. As the pathogen does not respect international boundaries, migration between regions is highly likely.

In eastern India the four major potato producing states are Assam, Bihar, Meghalaya, and West Bengal. These regions comprise approximately 45% of the total potato production in the country^33^. West Bengal is the second largest potato producing state after Uttar Pradesh. In 2014, a late blight epidemic in this region, primarily in West Bengal, led to dramatic social upheaval, including farmers committing suicide due to crop losses and policy changes such as setting minimum export values on potatoes^28^. During the epidemic year, the potato yield was approximately 8000 kg/ hectare less than what is expected for an average production year. A close examination of the pathogen populations during recent epidemics could provide insight into the source of *P*. *infestans* populations in India and spread within the country and help guide Indian growers to more effective disease management practices.

The objectives of this study were to (i) analyse the phenotypic and genotypic variation of the *P*. *infestans* population causing late blight in different regions of India (ii) develop a baseline of the current *P*. *infestans* genotypes in major potato growing regions for future studies and (iii) examine possible migration pathways for the pathogen in eastern and northeastern regions of India.

## Results

### Phenotypic and genotypic diversity

**Figure 1:**
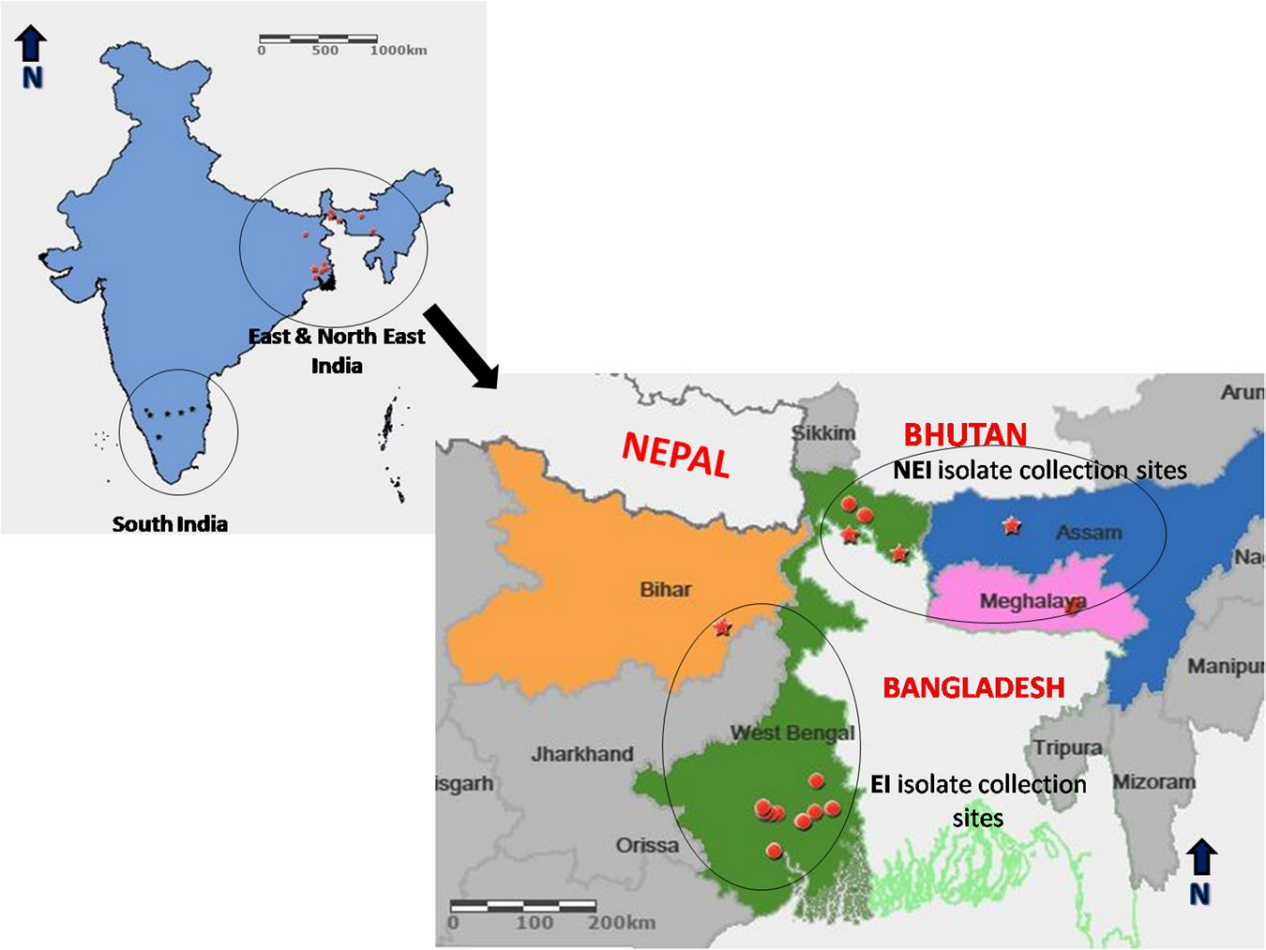
Geographical locations for sample collections and populations corresponding to the potato and tomato growing regions. Eastern India (EI), northeastern India (NEI) and southern India (SI) populations have been depicted. EI & NEI were sampled in this study. SI was collected previously^32^, was used in data analysis. Only major sites have been shown; stars depict Tomato and dots Potato hosts. Map was constructed by using ArcGIS online^78^ (http://www.esri.com/software/arcgis/arcgisonline).

A total of 59 isolates consisting of 19 from tomato and 40 from potato were collected between 2013–2014 growing season (Severe Late blight epidemic year) from the major potato and tomato growing regions (Fig. 1) (see Supplementary Table S1). All were of the A2 mating type and the Ia mtDNA haplotype. In addition, 57 of 59 isolates were resistant to metalaxyl, one was intermediately resistant, and one was sensitive (see Supplementary Table S1).

All the isolates were identified as the 13_A2 genotype on the basis of the RG57 probe RFLP fingerprint, the Ia mtDNA haplotype and the 12-plex SSR data^23,34^ (see Supplementary Table S2, see Supplementary Table S3). The SSRs also revealed mutations generating a third allele at the three most variable loci (D13, G11 and SSR4), allowing isolate discrimination and suggesting the clone was triploid.

### Population differentiation

**Figure 2:**
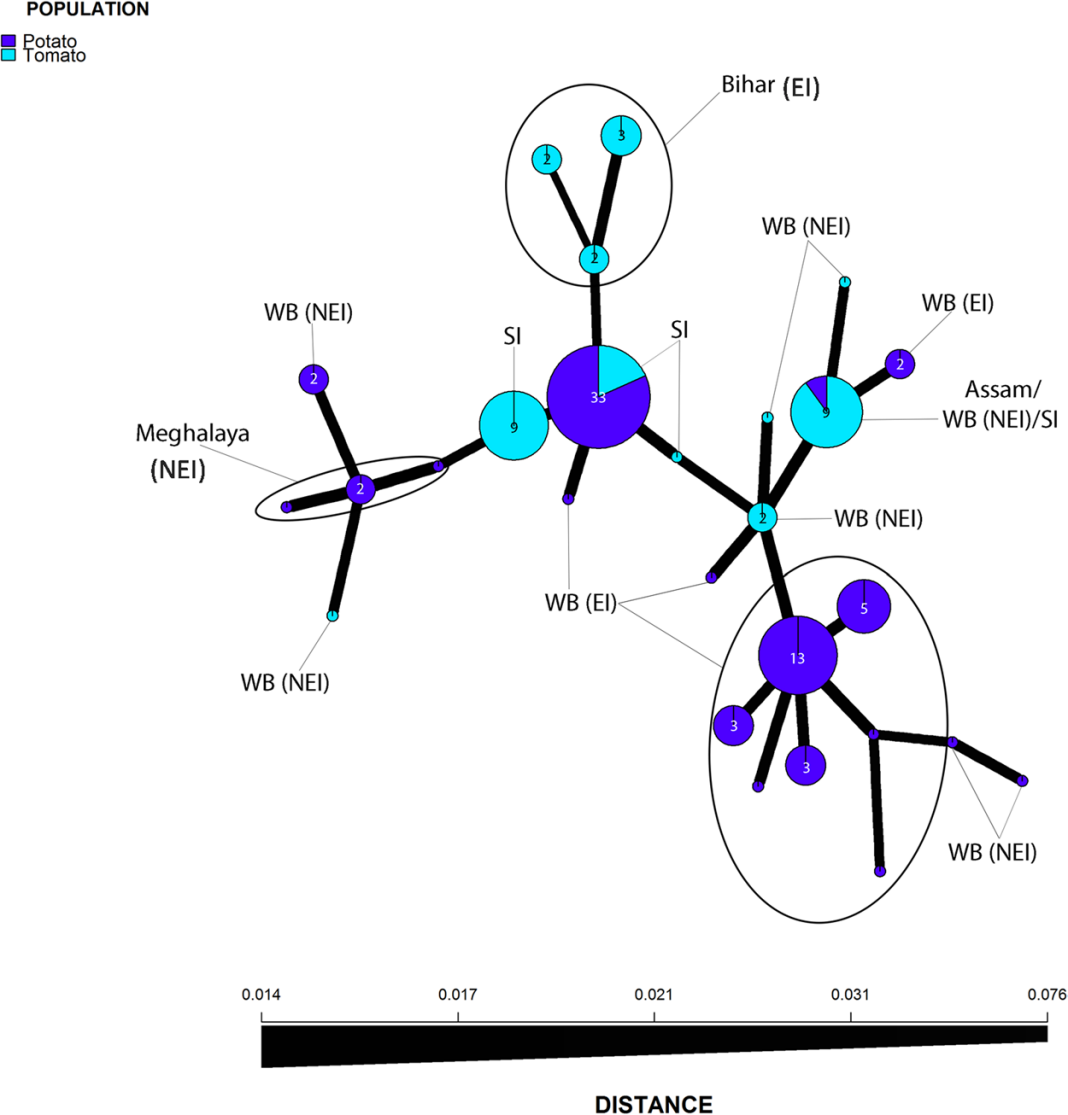
Minimum spanning network (MSN) of multilocus genotypes of *Phytophthora infestans* from populations in India based on host. The size of the node is proportional to the number of individuals within the multilocus genotype.

Examination of the population structure of *P*. *infestans* from Eastern India (EI) and North Eastern India (NEI) using SSRs identified a total of 24 multilocus genotypes (MLGs) amongst the 59 isolates. When another 45 isolates from south India (SI)^32^ were incorporated, a total 27 MLGs were identified amongst 104 isolates from India. No single MLG dominated in the EI and NEI population; the three most frequently sampled MLGs were found 13, 9, and 5 times (Fig. 2). Little difference was observed between the diversity of MLGs measured in EI and NEI populations (Table 1). Multilocus genotypes in both regions showed the same level of evenness (E5) and clonality based on Ia and ${\bar{r}}_{{\boldsymbol{d}}}\,$. The Ia and ${\bar{r}}_{{\boldsymbol{d}}}\,$ values were found to be significantly different from 0 based on 999 permutations, providing evidence in support of the presence of a clonal population (see Supplementary Table S4 and Supplementary Fig. S5). Due to its geography samples from the state of West Bengal (Fig. 1) were ascribed to Eastern and Northeastern subpopulations termed, WB-EI and WB-NEI, respectively. The WB-EI and WB-NEI samples were found to be the most diverse, with the greatest number of MLGs and a higher diversity index than other regions. Isolates collected from Assam were found to be the least diverse, with only one MLG observed (Table 1). A total of 38 alleles were identified across 12 loci with a mean of 3.17 (Table 2). Among these, the D13 locus had the most alleles, followed by PinfSSR4, PiG11, and Pi4B. Loci Pi70, PinfSSR11, and PinfSSR2 were the least diverse with 1 allele each. Of loci with more than two alleles, Pi4B was found to be the most evenly distributed (Table 2). In EI and NEI region isolates collected from potato were more diverse than isolates collected from tomato (Table 3). However, this finding may be due to the greater number of isolates collected from potato. When south Indian isolates^32^ were incorporated into the analysis, almost equal diversity were identified on both hosts in India (see Supplementary Table S6)

**Table 1:** Diversity statistics for microsatellite data for 12 microsatellite loci in populations of *Phytophthora infestans* in India by region and by state.

Population	*N* ^a^	MLG^b^	eMLG(SE) ^c^	H^d^	Hexp^e^	Evenness	Ia^f^	${\bar{r}}_{{\boldsymbol{d}}}^{g}\,$
**Region**								
Eastern India	38	13	9.93(1.18)	2.18	0.426	0.647	0.997	0.238
Northeastern India	21	11	11(0)	2.05	0.432	0.634	0.947	0.236
All	59	24	12.90(1.61)	2.79	0.428	0.647	0.770	0.152
**State**								
Eastern West Bengal	31	10	5.45(1.092)	1.84	0.423	0.631	0.0967	0.030
Northeastern West Bengal	12	8	7.14(0.649)	1.98	0.445	0.889	0.9001	0.210
Assam	5	1	1(N/A)	0.00	0.417	N/A	N/A	N/A
Bihar	7	3	3(N/A)	1.08	0.409	0.969	0.7439	0.372
Meghalaya	4	3	3(N/A)	1.04	0.468	0.912	−0.333	−0.333
South India	45	4	2.54(0.672)	0.772	0.388	0.621	−0.0728	−0.0834
All	104	27	6.55(1.294)	2.55	0.42	0.515	0.9012	0.1944

^a^*n*: number of individuals.

^b^MLG: number of multilocus genotypes (MLG).

^c^eMLG: expected number of MLGs at smallest size of at least ten; SE: Standard error.

^d^H: Shannon-Weiner Index of MLG diversity.

^e^Hexp: Nei’s 1978 expected heterozygosity.

^f^Ia: Index of Association.

^g^${\bar{r}}_{d}\,$: standardized index of association.

**Table 2:** Population statistics for clone corrected microsatellite data for 12 microsatellite loci in populations of *Phytophthora infestans* in India.

Locus	*n* ^a^	1-D^b^	Evenness
Pi02	2	0.50	1
Pi4B	3	0.51	0.95
PiG11	6	0.63	0.78
Pi04	2	0.50	1
Pi63	2	0.50	1
Pi70	1	N/A	N/A
D13	9	0.72	0.67
PinfSSR11	1	N/A	N/A
PinfSSR2	1	N/A	N/A
PinfSSR4	7	0.66	0.80
PinfSSR6	2	0.50	1
PinfSSR8	2	0.50	1
Mean	3.17	0.42	0.91

^a^*n*: number of individuals (not clone corrected).

^b^1-D: Simpson index.

**Table 3:** Diversity statistics for all 12 loci in Eastern and North Eastern Indian populations of *Phytophthora infestans* based on host.

Population	*N* ^a^	MLG^b^	eMLG(SE) ^c^	H^d^	Hexp^e^	Evenness	Ia^f^	${\bar{{\boldsymbol{r}}}}_{{\boldsymbol{d}}}^{{\boldsymbol{g}}}\,$
Original								
Potato	40	17	10.6(1.41)	2.39	0.434	0.592	0.748	0.224
Tomato	19	8	8(0)	1.84	0.411	0.749	0.922	0.183
All	59	24	12(1.57)	2.79	0.428	0.647	0.770	0.152

^a^*n*: number of individuals (not clone corrected).

^b^MLG: number of multilocus genotypes (MLG).

^c^eMLG: expected number of MLGs at smallest size of at least ten; SE: Standard error;

^d^H: Shannon-Weiner Index of MLG diversity.

^e^Hexp: Nei’s 1978 expected heterozygosity.

^f^Ia: Index of Association.

^g^ $\bar{r}\,$_*d*_: standardized index of association.

The minimum spanning network (MSN) shows that no MLGs were shared between the eastern and northeastern regions, though MLGs from both regions were interspersed throughout the network (Fig. 2). Examination of the MSN based on host indicates that all except two of the ten MLGs from tomato were host-specific. One of the exceptions was from the NEI region where there was a single isolate from potato in the same MLG as eight isolates from tomato probably because the potato isolate was from a field adjacent to the infected tomato crop. The other, was a MLG composed of only south Indian isolates^32^.

In addition, one of the four MLGs detected in south India matched the MLGs of *P*. *infestans* from disease outbreaks sampled in eastern and northeast India in the current study (Fig. 2). An examination of MLG relatedness by region indicated a greater range of MLG diversity in West Bengal (both WB-EI and WB-NEI) than in Bihar and Meghalaya. However, as the administrative region of West Bengal (WB) covers a large geographic distance (Fig. 1). MLGs from WB-NEI and WB-EI were interspersed throughout the network. The cluster of three related MLGs each from Bihar and Meghalaya were distinct from other MLGs found in West Bengal. The five isolates from Assam, mostly from tomato, formed a single cluster along with three isolates from the neighbouring northern part of West Bengal from the northeastern population.

The lowest values of F_ST_ calculated were between the WB-EI and Assam populations (F_ST_ = 0.00673) and between the WB-NEI and Assam populations (F_ST_ = 0.00767). Conversely, the lowest level of gene flow was observed between Meghalaya and Assam (F_ST_ = 0.03680). These values are reflected in the number of migrants calculated for each population pair, with Assam and WB-EI/WB-NEI exhibiting the most number of migrants per generation (Nm = 24.607211 and 21.55849, respectively) and Assam and Meghalaya exhibiting the fewest (Nm = 4.36286) (Table 4).

**Table 4:** Pairwise calculations of F_ST_ (top diagonal) and Nm (bottom diagonal) for populations of *Phytophthora infestans* in multiple regions of India.

Region	Assam	Eastern West Bengal	Northeastern West Bengal	Meghalaya	Bihar	South India
Assam		0.00673	0.00767	0.03680	0.02819	0.00903
Eastern West Bengal	24.607211		0.00759	0.01327	0.02300	0.02973
Northeastern West Bengal	21.55849	21.80198		0.01012	0.02262	0.01396
Meghalaya	4.36286	12.39732	16.30388		0.03537	0.01065
Bihar	5.74591	7.77150	7.20171	4.54520		0.01321
South India	18.28453	5.43934	11.77300	15.48405	12.44742	

**Figure 3:**
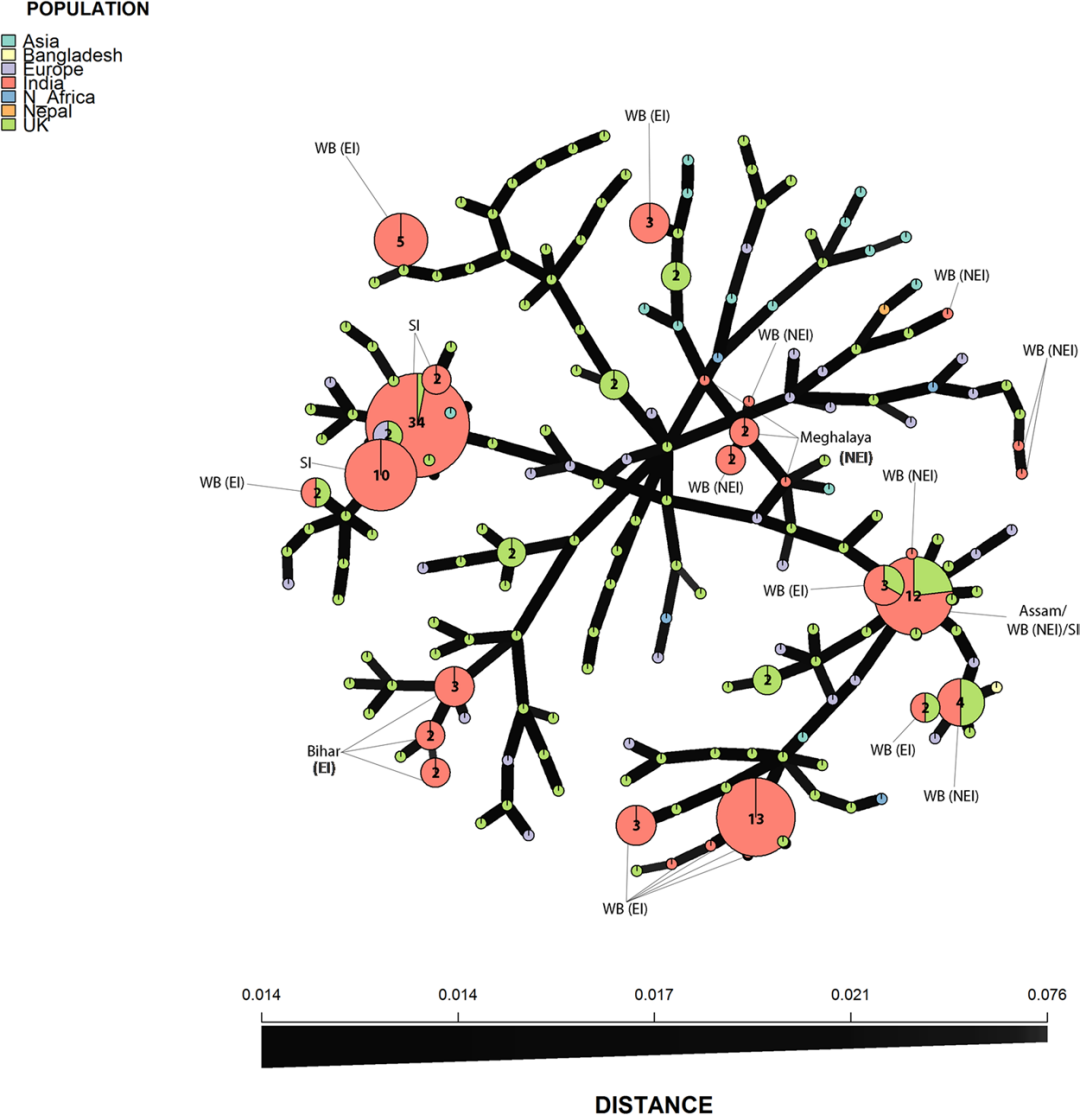
Minimum Spanning Network (MSN) of *Phytophthora infestans* 13_A2 MLGs from India compared to representative MLGs found amongst a global sample of isolates.

Nineteen of the twenty four 13_A2 variants identified in the sample from eastern and north eastern India were unique to this region (see Supplementary Table S3) when compared to a global sample of 172 13_A2 variants (see Supplementary Table S7). The structure analysis did not reveal any evidence for more than one population. The MSN of Indian and global populations indicated that MLGs of Indian *P*. *infestans* populations were dispersed across the network rather than forming a distinct single cluster (Fig. 3). Samples from West Bengal were the most diverse and grouped with MLGs found in Europe, the UK and from neighbouring Bangladesh but were not linked directly to most samples from south India. A large MLG (MLG20) comprising isolates from Assam, West Bengal and south India was shared with isolates previously found in the UK. Other samples tended to form discrete clusters such as those collected in Bihar, South India and Meghalaya. The two former regions had MLGs most similar to samples from Europe whereas the latter had closer genetic similarities with MLGs from Europe and Asia.

### Population virulence

**Figure 4:**
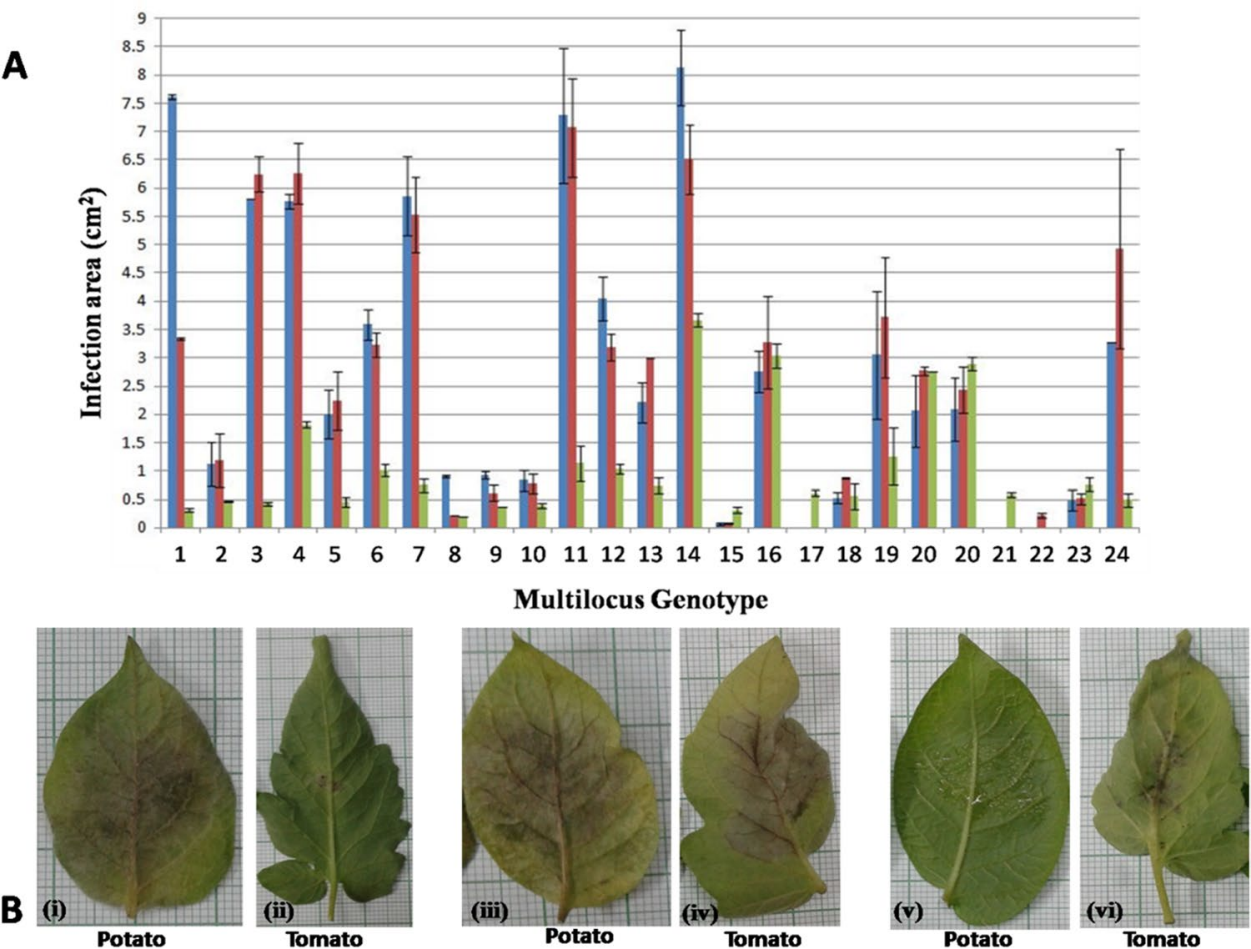
(**A**) The mean infection area of the 24 MLGs of 13_A2 lineage of *Phytophthora infestans* from eastern and northeastern India on two cultivars of potato (‘Kufri pukhraj’ &‘Kufri jyoti’) and one cultivar of tomato (‘Arka vikas’); shown as the blue, red and green bars, respectively. MLG20 had isolates from both potato and tomato and accordingly the MLG20 has been depicted twice here separately. (**B**) The corresponding pictures of the infection on potato and tomato in detached leaf assay; (i) and (ii) representative of MLGs more aggressive on potato, (iii) and (iv) equally aggressive on both hosts, (v) and (vi) no infection on potato.

Differences in the aggressiveness phenotype, based on lesion area, incubation period, and latent period, on potato and tomato hosts were noted amongst the tested isolates of each sub-clonal variant of the 13_A2 clonal lineage (Fig. 4). However, there was overall greater aggressiveness on both cultivars of potato than on tomato (*P value* < 0.01). The only exceptions were MLG16 and MLG20, which had almost equal aggressiveness on both hosts. No significant differences in aggressiveness on either host were observed based on the host of origin, but two MLGs (MLG17 and MLG21) from tomato, failed to infect potato, and one MLG from potato (MLG 22) did not infect tomato or the other ‘Kufri Pukhraj’ variety of potato. Differences in incubation period were also observed. MLGs 4, 5, and 6 had a two-day incubation period, while the rest had a three-day incubation period. All MLGs had a five-day latent period. However, the latent period for MLG 8 (tomato only), MLG 15 (potato only), and MLG 22 (Kufri Jyoti only) could not be determined, as no sporangia were seen after seven days of incubation (see Supplementary Table S8 and Fig. 4).

Analysis of *Avr3a* sequences identified the presence of the *Avr3a* EM type gene in EI and NEI populations. In addition, one synonymous (at base pair 363) and two nonsynonymous (at base pairs 43 and 370) mutations were also identified in the *Avr3a* EM type gene from fourteen isolates independently of their host of origin (see Supplementary Table S9). The polymorphism identified at position 370 leads to a change in the amino acid from arginine to glycine at position 124, located in the loop 3 region. Mutational analysis based on the model structure of Avr3a wild type (WT) predicted that the mutation with the highest likelihood to affect function at position 124 was glycine (see Supplementary Fig. S10).

## Discussion

The eastern region of India is the country’s largest potato growing region and was the site of a severe late blight epidemic in 2014^28^. DNA fingerprinting of 59 isolates of *P*. *infestans* identified 13_A2 (Blue_13), a European genotype, as the only clonal lineage causing the outbreaks. Sub-clonal variation in SSR fingerprints revealed some sub-structure in the local population that was linked to region and host. Most isolates from eastern and north-eastern India differed from global populations and those previously observed in southern India. Rapid changes in population structure, in which dominant lineages are replaced by newer, fitter, more aggressive lineages has been observed previously in *P*. *infestans*^16,35^ and the 13_A2 lineage is a modern example of this pattern. Since its first isolation in Germany and the Netherlands in 2004, the lineage has successfully replaced other genotypes and become dominant in parts of western Europe^23,36^. This lineage has subsequently spread to other parts of the world, including China and India^25,26^. Recent work from southern India reported that the importation of 13_A2 most likely on seed potatoes from the NL or the UK was responsible for severe outbreaks of late blight on tomato and potato^26,32^. Four sub-clonal MLGs were identified amongst 45 isolates in the south. In contrast, the populations from north-eastern and eastern India examined in our study were more diverse with 24 MLGs identified from both hosts, 19 of which were unique to the region. This means that multiple mechanisms were probably at play; not only new introductions of variants from the UK or /and Europe may have occurred, but also local mutations and adaptations took place as discussed in detail later on.

Recent work has confirmed that the 13_A2 lineage is triploid^37^ which is consistent with the observed high levels of heterozygosity and SSR polymorphism^23,25,36^. In this study, some loci such as D13, PiG11 and PinfSSR4 were highly diverse and others such as PinfSSR11, Pi70 and PinfSSR2 were monomorphic and homozygous. The eastern and north-eastern Indian populations examined here showed high levels of diversity with novel mutations, in particular at the loci D13, PinfSSR4 and PiG11, that generated 19 MLGs not reported previously (Fig. 3). Such mutations are helpful in tracking populations of *P*. *infestans* on local and global scales.

Evidence of phenotypic changes in fungicide sensitivity have also been documented in earlier studies which showed that in the plains of northern India, the A1 mating type and metalaxyl sensitive isolates, presumably US-1, were prevalent, but later the population shifted to a metalaxyl resistant populations even though the A1 mating type was still observed^38–40^. Though no evidence of sexual reproduction has been recorded in the eastern and northeastern parts of India, the A2 mating type was observed in Meghalaya^38^. All our isolates were found to be of the A2 mating type and all except two tomato isolates were metalaxyl resistant. Mitochondrial haplotyping indicated that all isolates were the Ia mt DNA haplotype. Previous reports indicated that the A1 isolates were either the Ia or Ib haplotype^38,41,42^. This study thus confirms the presence of the A2 mating type in this region and the absence of the A1 mating type suggests the new A2 population is aggressive and has displaced the former population. Such a displacement by 13_A2 is consistent with studies in Europe^23^. This aggressive and metalaxyl resistant pathogen population will make late blight more challenging to manage and it is important that local management decisions for growers should reflect this in these regions.

Aggressiveness and host preferences are key factors that affect disease outbreaks. For example, US lineages US-8 and US-24 predominantly infect potato, while US-22, the primary lineage during a major outbreak in the US in 2009, infects both potato and tomato^6,43^. Such host adaptation either on potato or tomato has also been reported elsewhere^19,44,45^. The 13_A2 lineages in southern India were equally aggressive on both hosts^32^. Our results show some variation in aggressiveness within the 13_A2 clonal lineages. Detached leaf assays showed the pathogen to be more aggressive on potato than tomato irrespective of host of origin. However, we identified two MLGs from tomato that did not infect potato and one MLG from potato that did not infect tomato or the ‘Kufri Pukhraj’ cultivar of potato (Fig. 4). Such differences in host specificity may drive the observed subdivision of the clonal population by host (Fig. 2). Only one of the eight isolates of MLG20, for example, was from potato and the potato field where this isolate was collected was adjacent to tomato fields where other isolates of this MLG were collected. Similarly the distinct sub-clones in Bihar all originated from tomato. These findings provide some evidence of hitherto unseen sub-clonal variation based on host within the 13_A2 clonal lineage. Other studies on Indian *P*. *infestans* isolates have shown there is no difference in aggressiveness and host specificity between tomato and potato isolates^32,40,46^. Further work needs to be done to explore this question.

The presence of the EM form of the AVR3a effector is consistent with its reported virulence against the R3a gene^23^. Additionally, the detection of two novel non-synonymous mutations at positions 15 (signal peptide) and 124 (loop 3) within the *avr3a* locus suggest these populations may be unique among current known 13_A2 populations. Moreover, since it is possible that the mutation identified at position 124 may affect the function of this protein, further characterization of these isolates is needed.

Many factors regulate pathogen population dynamics including environmental forces, selection pressure, new aggressive strains, increasing global trade, and host dynamics^20,47^. Eastern and north eastern India are areas where movement of host material affects pathogen populations and changes in pathogen population dynamics can lead to severe disease outbreaks. The annual mean temperature in this sub-tropical region remains around 30 °C and during the summer temperatures can reach up to 42 °C. Potato cultivation occurs mainly in paddy fields that are waterlogged for most of the year. During the monsoon season, flooding is also a recurring problem in major potato growing regions of West Bengal. Since the pathogen population is reproducing asexually, oospores are not present for overwintering. In addition, sporangia cannot survive beyond 20 days in waterlogged conditions^48^. Due to these adverse environmental factors each year and the practice of rotating rice cultivation (where the field is also submerged) there is an almost complete elimination of both host and pathogen which means populations of *P*. *infestans* face a narrow genetic bottleneck. The pathogen survives only in infected stored seeds kept by small shareholders and use of infected seed impacts pathogen dispersal between cropping seasons. This asexual vertical transmission between seasons is partially facilitated by growers planting moderately resistant tubers^47^ such as ‘Kufri Jyoti’ and ‘Kufri Pukhraj’ that are mainly cultivated in these regions. Since the tubers are not highly resistant to infection, less aggressive isolates with longer latent periods (e.g. those in our study with latent periods >7 days) are more likely to survive to spread, as they produce less severe symptoms. Thus, seed planted during the next growing season can survive^47^ and act as a source of inoculum. In this region, new founder populations emerge each year, persist for some weeks, and then disappear. This pattern is made possible by the fact that potato is mainly a seasonal winter crop across most of this region (except for hill regions like Darjeeling and Shillong). There is also a difference in the way the host material (i.e. seed potato) is used in these regions. Seed in West Bengal comes almost every two to three years mostly from northern states (like Punjab). In contrast, farmers in Meghalaya tend to use potato seed which they have stored from past years^49^. These differential cultural practices, environmental factors, crop rotation and grower behaviour can compound to influence pathogen diversity and prevalence^50^ and might explain the differences in observed genetic diversity of *P*. *infestans* in these regions.

Our data show that while the Indian late blight populations are clonal, there is more subclonal diversity within the 13_A2 lineage than previously reported. Since north eastern and eastern India shares international borders with Bangladesh and Nepal, both of which regularly import potatoes from outside sources, and because of the practice among small marginal farmers of storing seed to use for the next season, the diversity observed may be the result of both local migrations of the pathogen across borders and common local agricultural practices. This diversity may be exacerbated by a local weather phenomena called the ‘Western Disturbance’. This occurs in northern India, causing meteorological changes in eastern India and generating 5–6 day periods of wet, cool and windy weather suitable for *P*. *infestans* infection. The subsequent rapid dispersal and mixture of the rapidly expanding clonal populations due to this wind is likely to increase the diversity of *P*. *infestans* in this region.

Stepwise mutation of SSR loci generates variants that can be used to track local and migrant populations providing inferences on the spread of *P*. *infestans* inoculum via seed trade or airborne sporangial routes. The population structure of *P infestans* in India indicates that the 13_A2 lineage migrated recently to India likely due to movement of infected plant material from the UK and Europe^26^. Three probable pathways for interstate migration occur in India; one includes movement of *P*. *infestans* from the South Indian population upwards towards Bihar, the second from the bordering regions into West Bengal, possibly from neighboring countries like Bangladesh and Nepal. A third possibility includes direct import of infected tubers from Europe. The MSN for the 13_A2 population across the whole of India revealed 27 MLGs with some evidence of a regional sub-structure (Fig. 2). One isolate from southern India shared an MLG with those from the northeastern India (MLG20 from Assam and NEI-West Bengal) and these were all isolated from tomato^32^ which could support this as a migrant population. However, comparisons with the larger collection of 13_A2 variants (Fig. 3) showed this was also shared with the population in Europe and was an MLG defined early in the history of 13_A2 in Europe^23,36^. This MLG is thus equally likely to have been imported into northeastern India independently of the migrant pathogen population in the south. The other three MLGs from southern India were distinct from those in the current study also supporting an independent emergence of the population in the northeastern regions of India rather than a migration from the south. Only isolates from Bihar displayed a distinct local cluster and this was supported in comparisons with the wider sample of variants from global populations (Fig. 3). A comparison of the 13_A2 variants identified in a cross section of global populations with those from north eastern and eastern India is informative. The presence of Indian MLGs across the MSN suggests either multiple independent imports or the importation of highly diverse populations of *P*. *infestans* 13_A2 inoculum into the region. Some local clustering of northeast and eastern Indian populations from Bihar and Meghalaya is apparent but the variants from both West Bengal-EI and WB-NEI are scattered across the network. Shared allelic diversity with African and Asian isolates were noted but further more detailed analysis of greater sample numbers from these countries is required to determine those pathways of spread more precisely. It should also be noted that associations between MLG and location may be the result of chance mutations independently generating the same MLG. The resultant homoplasy can confound such analysis of dispersal pathways.

The data presented here can serve as a baseline for further studies of the diversity of Indian populations of *P*. *infestans* and for surveillance programs for improved management. The continued monitoring of these populations will provide forewarning of any new population shifts and potentially provide additional time to react in the face of another epidemic. India has yet to begin contributions to pathogen surveillance systems, including the Asiablight program^51^, which mirrors the objectives of the Euroblight program (http://euroblight.net/) and USAblight (http://USAblight.org). However, the results of our study can now contribute to forming baseline microsatellite data for an Indiablight network or a larger Asiablight-based monitoring system in India. Our data also point to the need for improved phytosanitary measures to curtail importation of infected seed potatoes into India. The history of seed trade between India and Europe suggests that improved seed certification may be needed to suppress the additional introduction of new strains of *P*. *infestans* into the country.

## Materials and Methods

### *Phytophthora infestans* collection, isolation and storage

A single symptomatic sporulating late blight lesion from foliage of potatoes, and leaflets and fruits of tomato, were collected from farmers’ fields in the major potato and tomato growing regions in eastern (EI) and north eastern India (NEI) during 2013–14 growing season. Each sample was carefully collected, placed in a plastic zipper bag, marked, and transported to the laboratory within 24 hours. The details for each *P*. *infestans* isolate collection are indicated in Supplementary Table S1.

Collected samples were processed using a routine *P*. *infestans* isolation protocol^52^. Small freshly sporulating leaf pieces (5 mm^2^) were then placed on top or into a selective medium [pea agar amended with rifamycin (20 mg/L), vancomycin (50 mg/L), ampicillin (100 mg/L), Polymixin B (50 mg/L), pentachloronitrobenzene (50 mg/L), and carbendazim (100 mg/L)]^53^. The plates were incubated in the dark at 18 °C for 5–10 days. *P*.*infestans* colonies were selected from these plates and transferred onto a fresh pea agar plate without any antibiotics^43^ for pure culture.

Cryopreservation: Agar plugs containing mycelium were suspended in 1 ml of cryoprotectant (10% glycerol) in screw-cap polypropylene vials, placed vials in ‘Mr. Frosty’ (Nalgene) tubes and then an uncontrolled cooling protocol^54^ was followed.

### Extraction of total DNA

Mycelial plugs from individual isolates were transferred to pea broth^53^. Mycelium from the 5 day old culture was harvested and excess medium was removed by filtration and washed in autoclaved distilled water. Approximately 0.1 g of mycelium was placed in a sterile 1.5 ml eppendorf tube and total DNA was extracted^55^.

### Determination of mating type

Mating type was determined using molecular markers. The markers for A1 and A2 mating type are - A1- INF-1, INF-2^56^, A2 - PHYB-1,PHYB-2^57^ primers were used. The validity of the marker method for testing mating type was tested using a set of control cultures of known mating type.

### Mitochondrial DNA haplotype and RG-57 fingerprinting

Mitochondrial DNA (mt DNA) haplotypes were identified using the PCR-RFLP methods^58^. DNA fingerprinting was carried out using the RG57 multilocus nuclear DNA probe^59^. Total DNA from isolates along with a standard US-1 and 13_A2 (2006_3928A) isolate were used as standard.

### Multiplexed microsatellite marker analysis

*P*. *infestans* simple sequence repeat (SSR) loci were genotyped using a modified version of the protocol for 12-plex single sequence repeat genotyping as described previously^34^. The *P*. *infestans* isolates were run alongside a standard 13_A2 sample (2006_3928A). The Qiagen Type-It Microsatellite PCR kit (Qiagen Corporation, Valenica CA) was used for PCR reactions, and sample volumes were modified to run a 12.5 µL reaction by using 6.25 µL 2× Type-It Master Mix, 1.25 µL of a 10× multiplex primer master mix, 4 µL PCR grade water, and 1–2 µL of template DNA (5–10 ng). Thermal cycling conditions as described earlier^60^. Fragments were analyzed on an Applied Biosystems 3730xl DNA analyzer. The peak size was determined against a GeneScan 500 LIZ standard and alleles were scored manually using Peak Scanner 2 (Applied Biosystems, Foster City, CA), and fragment lengths were rounded to the nearest whole number for analysis.

### Metalaxyl sensitivity

Sensitivity to metalaxyl was determined using an agar technique^52^. Pea agar plates amended with 5 and 100 ppm of metalaxyl (Glazer 35 WS®, Rallis India Ltd.) were prepared. Agar blocks (8 mm diameter) with actively growing mycelia were taken from the colony margin of each isolate and transferred to the centre of three replicate plates of metalaxyl-amended and non amended (served as control) pea agar plates. After 7 days of incubation at 18 °C in dark the metalaxyl sensitivity were checked as described earlier^52^. The metalaxyl sensitivity experiment was replicated twice for each isolate.

### Virulence and infection area determination

A total of 25 isolates, one representative isolate from each MLG and two representative isolates from MLG 20 (one isolate from potato and one isolate from tomato), were selected for virulence and aggressiveness studies. Isolates were tested following the detached leaf assay on two cultivars of potato, Kufri Pukhraj and Kufri Jyoti, both classified as moderately resistant to late blight^61,62^ and on the tomato cultivar Arka Vikas, which has no known resistance genes^32^. Tomato and potato plants were grown in a glasshouse in plastic pots. The plants were maintained at a mean daily temperature of 22–23 °C under a 16-h day photoperiod. Uniform leaflets of 6-week-old potato and tomato plants were harvested and placed, abaxial side up, on moist sterile filter paper in 90 mm sterile Petri dishes.

A sporangial suspension of a *P*. *infestans* isolate of each multi locus genotype (MLG) was prepared by scraping the surface of 15-day-old pea agar cultures in sterile distilled water. The concentration of the resulting suspension was determined with a haemocytometer and adjusted to 1 × 10^4^ sporangia mL^–1^. The suspension was maintained at 4 °C for 2 h before inoculation. For inoculation, three leaflets were used per sample. Each leaflet was inoculated by placing one 20 μL drop of a sporangial suspension on the abaxial surface near the midrib and incubated for 7 days at 18 °C with a light and dark cycle of 16 and 8 hours respectively as described earlier^32,43,63,64^. Plates were placed in a completely randomised design. The full experiment was repeated twice independently.

The virulence and aggressiveness of 25 isolates representing each MLG were evaluated daily after inoculation and incubation period (IP) and latent period (LP) were determined^65^. After 7 days of incubation, the infected leaflets were placed on 1 mm^2^ graph paper and photographed. The lesion area was measured by using Image J software^66^. The measured lesion areas of both hosts (potato and tomato) were checked statistically to compare the aggressiveness of each MLGs and determine the significance of the effects of host of origin, target host and MLGs. For this, a non-parametric method of Wilcoxon rank sum test for comparing among groups were used^67^.

### Avr3a gene sequencing and annotation

The Avr3a gene was amplified using the primers with a M13 tail to allow for amplification and sequencing of 453 bp corresponding to the entire gene^68^. Single band PCR products were further purified and sequenced for both strands by a commercial service (Xcelris labs Ltd, India). The sequences were then aligned by using tools available in Eumicrobe DB^69^ and again compared manually with the supplied electropherogram data. A blast search was performed for each strand to compare the Avr3a sequences generated with those available in GenBank. Polymorphic positions were further analysed by using PHYRE2 investigator^70^ to check which base has the highest likelihood of affecting function at respective polymorphic positions.

### Data analysis

MLGs identified using SSRs were evaluated at regional, state, and global levels. At the regional level, data were partitioned into northeastern (NEI) and eastern (EI) regions for comparison. At the state level, analysis included haplotypic SSR data from southern India (SI)^32^. Due to the geographic distance between collection sites in West Bengal, the state was divided into WB-EI and WB-NEI. All SSR data collected from India were compared to a global set of single representative samples of 13_A2 variants. To ensure consistent comparisons peak calls were calibrated to the allele naming scheme^34^. Allele sizes were also compared to other data sets from India^32^.

Analysis of SSR genotypes was conducted using the program Structure v.2.3.4^71^. The data were run using a 20,000 repeat burn-in and 1,000,000 MCMC repeats under both an admixture model and a model assuming no admixture. Structure was allowed to incorporate population data into the analysis (LOCPRIOR) as this setting can be used to discern more subtle population differences. Independent runs of the model used *K* values from 1 to 10 with 20 replicate runs at each value of *K*. The optimal *K* was estimated using the Evanno method in the web tool Structure Harvester^72^. In addition, the optimal K was inferred through direct observation of groupings of the samples by their estimated likelihoods (Ln P (D)). All runs for the optimal K values, as well as surrounding non-optimal K values, were averaged using CLUMPP v. 1.1.2^73^ and visualized with the program Distruct v. 1.1^74^.

Locus statistics and population statistics were generated using the R library *poppr*^75^. Clone correction was performed as needed using the clonecorrect () function in *poppr*. Clone correction reduces the number of individuals in each population to one representative per MLG. *Poppr* was also utilized to generate minimum spanning networks (MSN) for MLGs at each spatial level. At the regional level, the index of association (Ia) and the standardized index of association (${\bar{r}}_{{\boldsymbol{d}}}\,$) were calculated and evaluated for significance using 999 permutations for both clone corrected and non clone corrected data. *Polysat*^76^ was used at the state level to calculate pairwise fixation indices (F_ST_). Since the amount of selfing in the system is unknown, a simple frequency calculator in *polysat* was used to generate allele frequencies for the calculation of F_ST_. It should be noted that this calculator assumes all alleles have the same chance to be present in more than one copy, and as such may result in an underestimated F_ST_. For the purposes of the calculation, the populations were assumed to be autopolyploid. F_ST_ values were converted to the number of migrants per generation (Nm) using the R library *StrataG*^77^. A ploidy of 3 was assumed for the calculation.

### Data Availability

All data generated or analysed during this study are included in this published article (and its Supplementary Information files). Maps in Fig. 1 created using ArcGIS® online by Esri. ArcGIS® and ArcMap™ are the intellectual property of Esri and are used herein under trial version. Copyright^©^ Esri. All rights reserved. For more information about Esri® software, please visit www.esri.com.

## Electronic supplementary material

Supplementary information

Supplementary Table S7

Supplementary Table S9
