# Supplementary material for: Large sub-clonal variation in Phytophthora infestans from recent severe late blight epidemics in India
Source: Sci Rep. 2018 Mar 13;8:4429. doi: 10.1038/s41598-018-22192-1 (PMC5849725; doi:10.1038/s41598-018-22192-1)
Supplement: Supplementary file 1 — Supplementary information [file 41598_2018_22192_MOESM1_ESM.doc]

**Large sub-clonal variation in *Phytophthora infestans* from recent severe late blight epidemics in India**

Tanmoy Dey^1^, Amanda Saville^2^, Kevin Myers^3^, Susanta Tewari^4^, David E. L. Cooke^5^, Sucheta Tripathy^6,7^, William E. Fry^3^, Jean B. Ristaino^2^, Sanjoy Guha Roy^1^*

^1^Department of Botany, West Bengal State University, Kolkata 700126, India. ^2^Department of Entomology and Plant Pathology, North Carolina State University, Raleigh, North Carolina, Raleigh, NC 27607, USA. ^3^Plant Pathology and Plant-Microbe Biology, Cornell University, Ithaca, NY14850, USA. ^4^TeraBundle Anlytics Pvt. Ltd. 721602. India ^5^James Hutton Institute, Dundee, DD2 5DA, UK. ^6^Indian Institute of Chemical Biology, Kolkata 700032, India.^7^Academy of Scientific and Innovative Research, Ghaziabad, Delhi, India.

* Corresponding author: s_guharoy@yahoo.com

| **Isolate Name** | **Multilocus genotype** | **Host Name and Cultivar** | **State** | **Region** | **Latitude** | **Longitude** | **Mating type** | **Mitochondrial Haplotype** | **Metalaxyl sensitivity^a^** |
| --- | --- | --- | --- | --- | --- | --- | --- | --- | --- |
| Chamurchi jyoti 2 | 1 | Potato  Kufri jyoti | West Bengal | NEI | 26°34' 34.9'' N, | 88° 58'12.3'' E | A2 | Ia | R |
| Lataguri serpa alu 2 | 8 | Potato  Kufri sherpa | West Bengal | NEI | 26° 42' 52.2'' N | 88° 45'40.8''E | A2 | Ia | R |
| Chamurchi jyoti 1 | 1 | Potato  Kufri jyoti | West Bengal | NEI | 26°34'20.2''N | 88°58'35.6''E | A2 | Ia | R |
| Haldibari F-2 | 19 | Tomato Rocky | West Bengal | NEI | 26°20'48.5''N | 88°46'11.4''E | A2 | Ia | R |
| Nadia chandirampur 1 | 11 | Potato  Kufri jyoti | West Bengal | EI | 22°59'30.2''N | 88°31'54.9''E | A2 | Ia | R |
| Haldibari F-1 | 17 | Tomato Rocky | West Bengal | NEI | 26° 20' 57.0'' N | 88° 46'29.2'' E | A2 | Ia | I |
| Haldibari F-3 | 19 | Tomato Rocky | West Bengal | NEI | 26°20'39.1''N | 88°46'46.4''E | A2 | Ia | R |
| Nadia F1 (2) | 11 | Potato  Kufri jyoti | West Bengal | EI | 22°59'50.9''N | 88°32'22.7''E | A2 | Ia | R |
| Lataguri serpa alu 1 | 9 | Potato  Kufri sherpa | West Bengal | NEI | 26°42'59.5''N | 88°45'42.9''E | A2 | Ia | R |
| Haldibari Tomato 5 | 7 | Tomato Rocky | West Bengal | NEI | 26°21'44.0''N | 88°46'11.2''E | A2 | Ia | R |
| Polba sangrampur F1S6 | 14 | Potato  Super six | West Bengal | EI | 22°57'33.0''N | 88°18'47.4''E | A2 | Ia | R |
| Nadia F-2 | 11 | Potato  Kufri jyoti | West Bengal | EI | 22°59'58.4''N | 88°32'32.9''E | A2 | Ia | R |
| Sarisha Pukhraj 1 | 14 | Potato  Kufri pukhraj | West Bengal | EI | 23°00'28.6''N | 87°36'44.4''E | A2 | Ia | R |
| Modina jyoti 4 | 14 | Potato  Kufri jyoti | West Bengal | EI | 22°55'36.1''N | 87°42'25.7''E | A2 | Ia | R |
| Panahar 1 | 14 | Potato Kufri pukhraj | West Bengal | EI | 22°59'18.9''N | 87°36' 0.6''E | A2 | Ia | R |
| Modina jyoti 3 | 14 | Potato  Kufri jyoti | West Bengal | EI | 22°55'43.3''N | 87° 47' 31.1'' E | A2 | Ia | R |
| Bonmukha jyoti | 13 | Potato  Kufri jyoti | West Bengal | EI | 22°59' 19.8'' N | 87°37' 55.9'' E | A2 | Ia | R |
| panahar 3 | 14 | Potato  Kufri pukhraj | West Bengal | EI | 22°59'11.1''N | 87°35'46.2''E | A2 | Ia | R |
| DishraS6 1 | 24 | Potato  Super six | West Bengal | EI | 22°56'15.6''N | 87°36' 51.7''E | A2 | Ia | R |
| DishraS6 2 | 3 | Potato  Super six | West Bengal | EI | 22°56'37.7''N | 87°36'33.5''E | A2 | Ia | R |
| DishraS6 3 | 14 | Potato  Super six | West Bengal | EI | 22°56'56.6''N | 87°36'56.9''E | A2 | Ia | R |
| Maliya jyoti-1 | 10 | Potato  Kufri jyoti | West Bengal | EI | 22°49'59.8''N | 88°07'58.9E | A2 | Ia | R |
| Haripal 103 (1) | 14 | Potato Unknown cultivar | West Bengal | EI | 22°49'41.2''N | 88°08'35.1''E | A2 | Ia | R |
| Haripal S6 1 | 2 | Potato  Super six | West Bengal | EI | 22°50'01.6''N | 88°07'45.0''E | A2 | Ia | R |
| Maliya jyoti-2 | 18 | Potato Kufri jyoti | West Bengal | EI | 22°49'49.7''N | 88°08'24.3''E | A2 | Ia | R |
| Samudragarh stem B | 22 | Potato Kufri jyoti | West Bengal | EI | 23° 20' 26.4'' N | 88° 19' 30.7'' E | A2 | Ia | R |
| Modina joyti 1 | 14 | Potato Kufri jyoti | West Bengal | EI | 22°55'30.3''N | 87°43'02.4''E | A2 | Ia | R |
| DishraS6 4 | 3 | Potato  Super six | West Bengal | EI | 22°56'56.9''N | 87°37'11.7''E | A2 | Ia | R |
| Sarisha jyoti 2 | 14 | Potato  Kufri jyoti | West Bengal | EI | 23°00'34.0''N | 87°36'33.7''E | A2 | Ia | R |
| Haripal 103 (2) | 14 | Potato unknown cultivar | West Bengal | EI | 22°50'05.6''N | 88°08'43.0''E | A2 | Ia | R |
| Polba sangrampur F2S6 | 14 | Potato  Super six | West Bengal | EI | 22°57'15.1''N | 88°18'08.0''E | A2 | Ia | R |
| Mylliyam 1 | 4 | Potato local cultivar | Meghalaya | NEI | 25°30'16.2"N | 91°48'25.5"E | A2 | Ia | R |
| Mylliyam other side | 6 | Potato local cultivar | Meghalaya | NEI | 25°30'45.6"N | 91°47'04.8"E | A2 | Ia | R |
| Mawphlang jyoti | 4 | Potato  Kufri jyoti | Meghalaya | NEI | 25°26'53.8"N | 91°45'27.9"E | A2 | Ia | R |
| Mylliyam jyoti | 5 | Potato  Kufri jyoti | Meghalaya | NEI | 25°30'18.4"N | 91°48'29.7''E | A2 | Ia | R |
| HaldibariF1 (2) | 21 | Tomato unknown cultivar | West Bengal | NEI | 26°20'57.0''N | 88°46'29.2''E | A2 | Ia | R |
| Haripal 103 (3) | 14 | Potato Unknown cultivar | West Bengal | EI | 22°50'03.1''N | 88°08'51.4''E | A2 | Ia | R |
| Samudragarh D | 2 | Potato  Kufri jyoti | West Bengal | EI | 23°20'30.7"N | 88°19'30.5"E | A2 | Ia | R |
| Sarisha Pukhraj 2 | 3 | Potato  Kufri pukhraj | West Bengal | EI | 23°00'31.7"N | 87°36'35.5"E | A2 | Ia | R |
| Dinhata Tomato 1 | 20 | Tomato unknown cultivar | West Bengal | NEI | 26°08'0.45"N | 89°26'39.9"E | A2 | Ia | R |
| Dinhata Tomato 2 | 20 | Tomato unknown cultivar | West Bengal | NEI | 26°07'59.1"N | 89°26'13.1"E | A2 | Ia | R |
| Dinhata Tomato 3 | 20 | Tomato unknown cultivar | West Bengal | NEI | 26°07'58.0"N | 89°26'11.6"E | A2 | Ia | R |
| Barpeta Tomato 1 | 20 | Tomato unknown cultivar | Assam | NEI | 26°27'15.2"N | 90°57'59.7"E | A2 | Ia | R |
| Barpeta Tomato 2 | 20 | Tomato unknown cultivar | Assam | NEI | 26°27'15.6"N | 90°57'59.1"E | A2 | Ia | S |
| Barpeta Tomato 3 | 20 | Tomato unknown cultivar | Assam | NEI | 26°27'15.3"N | 90°58'00.4"E | A2 | Ia | R |
| Barpeta Tomato 4 | 20 | Tomato unknown cultivar | Assam | NEI | 26°27'15.3"N | 90°57'59.2"E | A2 | Ia | R |
| Barpeta Potato 2 | 20 | Potato unknown cultivar | Assam | NEI | 26°27'20.0"N | 90°57'59.7"E | A2 | Ia | R |
| Panskura 1 | 12 | Potato Kufri jyoti | West Bengal | EI | 22°28'8.3''N | 87°44'58.5''E | A2 | Ia | R |
| Panskura 2 | 12 | Potato Kufri jyoti | West Bengal | EI | 22°28'11.8"N | 87°44'54.7"E | A2 | Ia | R |
| Panskura 3 | 12 | Potato Kufri jyoti | West Bengal | EI | 22°28'10.8"N | 87°44'57.0"E | A2 | Ia | R |
| Panskura 4 | 12 | Potato Kufri jyoti | West Bengal | EI | 22°28'02.4"N | 87°45'06.3"E | A2 | Ia | R |
| Panskura 5 | 12 | Potato Kufri jyoti | West Bengal | EI | 22°28'05.5"N | 87°45'06.3"E | A2 | Ia | R |
| Sabour 2 | 23 | Tomato unknown cultivar | Bihar | EI | 25°14'0.2"N | 87°02'48.4"E | A2 | Ia | R |
| Sabour 3 | 23 | Tomato unknown cultivar | Bihar | EI | 25°14'02.5"N | 87°02'47.8"E | A2 | Ia | R |
| Sabour 4 | 16 | Tomato unknown cultivar | Bihar | EI | 25°14'02.5"N | 87°02'47.8"E | A2 | Ia | R |
| Sabour 6 | 16 | Tomato unknown cultivar | Bihar | EI | 25°14'01.4"N | 87°02'47.0"E | A2 | Ia | R |
| Sabour 7 | 15 | Tomato unknown cultivar | Bihar | EI | 25°14'01.4"N | 87°02'47.0"E | A2 | Ia | R |
| Sabour 8 | 16 | Tomato unknown cultivar | Bihar | EI | 25°14'00.9"N | 87°02'47.1"E | A2 | Ia | R |
| Sabour 9 | 15 | Tomato unknown cultivar | Bihar | EI | 25°14'00.9"N | 87°02'48.6"E | A2 | Ia | R |

**Supplementary Table S1 online:** Collection details and characteristics of *Phytophthora infestans* isolates of eastern and northeastern India in 2014.

^a^Metalaxyl sensitivity is indicated as follows: R: Resistant to metalaxyl; I: Intermediate sensitivity to metalaxyl; S: Sensitive to metalaxyl

EI- Eastern India, NEI- North Eastern India.

|  | **RG57 profile** | | | | | | | | | | | | | | | | |
| --- | --- | --- | --- | --- | --- | --- | --- | --- | --- | --- | --- | --- | --- | --- | --- | --- | --- |
| **Isolate Name** | 1 | 2 | 3 | 5 | 7 | 8 | 9 | 10 | 13 | 14 | 16 | 19 | 20 | 21 | 22 | 24 | 25 |
| Chamurchi jyoti 2 | 1 | 1 | 0 | 0 | 0 | 1 | 0 | 1 | 1 | 1 | 1 | 1 | 1 | 1 | 1 | 1 | 1 |
| Lataguri serpa alu 2 | 1 | 1 | 0 | 0 | 0 | 1 | 0 | 1 | 1 | 1 | 1 | 1 | 1 | 1 | 1 | 1 | 1 |
| Chamurchi jyoti 1 | 1 | 1 | 0 | 0 | 0 | 1 | 0 | 1 | 1 | 1 | 1 | 1 | 1 | 1 | 1 | 1 | 1 |
| Haldibari F-2 | 1 | 1 | 0 | 0 | 0 | 1 | 0 | 1 | 1 | 1 | 1 | 1 | 1 | 1 | 1 | 1 | 1 |
| Nadia chandirampur 1 | 1 | 1 | 0 | 0 | 0 | 1 | 0 | 1 | 1 | 1 | 1 | 1 | 1 | 1 | 1 | 1 | 1 |
| Haldibari F-1 | 1 | 1 | 0 | 0 | 0 | 1 | 0 | 1 | 1 | 1 | 1 | 1 | 1 | 1 | 1 | 1 | 1 |
| Haldibari F-3 | 1 | 1 | 0 | 0 | 0 | 1 | 0 | 1 | 1 | 1 | 1 | 1 | 1 | 1 | 1 | 1 | 1 |
| Nadia F1 (2) | 1 | 1 | 0 | 0 | 0 | 1 | 0 | 1 | 1 | 1 | 1 | 1 | 1 | 1 | 1 | 1 | 1 |
| Lataguri serpa alu 1 | 1 | 1 | 0 | 0 | 0 | 1 | 0 | 1 | 1 | 1 | 1 | 1 | 1 | 1 | 1 | 1 | 1 |
| Haldibari Tomato 5 | 1 | 1 | 0 | 0 | 0 | 1 | 0 | 1 | 1 | 1 | 1 | 1 | 1 | 1 | 1 | 1 | 1 |
| Polba sangrampur F1S6 | 1 | 1 | 0 | 0 | 0 | 1 | 0 | 1 | 1 | 1 | 1 | 1 | 1 | 1 | 1 | 1 | 1 |
| Nadia F-2 | 1 | 1 | 0 | 0 | 0 | 1 | 0 | 1 | 1 | 1 | 1 | 1 | 1 | 1 | 1 | 1 | 1 |
| Sarisha Pukhraj 1 | 1 | 1 | 0 | 0 | 0 | 1 | 0 | 1 | 1 | 1 | 1 | 1 | 1 | 1 | 1 | 1 | 1 |
| Modina jyoti 4 | 1 | 1 | 0 | 0 | 0 | 1 | 0 | 1 | 1 | 1 | 1 | 1 | 1 | 1 | 1 | 1 | 1 |
| Panahar 1 | 1 | 1 | 0 | 0 | 0 | 1 | 0 | 1 | 1 | 1 | 1 | 1 | 1 | 1 | 1 | 1 | 1 |
| Modina jyoti 3 | 1 | 1 | 0 | 0 | 0 | 1 | 0 | 1 | 1 | 1 | 1 | 1 | 1 | 1 | 1 | 1 | 1 |
| Bonmukha jyoti | 1 | 1 | 0 | 0 | 0 | 1 | 0 | 1 | 1 | 1 | 1 | 1 | 1 | 1 | 1 | 1 | 1 |
| panahar 3 | 1 | 1 | 0 | 0 | 0 | 1 | 0 | 1 | 1 | 1 | 1 | 1 | 1 | 1 | 1 | 1 | 1 |
| DishraS6 1 | 1 | 1 | 0 | 0 | 0 | 1 | 0 | 1 | 1 | 1 | 1 | 1 | 1 | 1 | 1 | 1 | 1 |
| DishraS6 2 | 1 | 1 | 0 | 0 | 0 | 1 | 0 | 1 | 1 | 1 | 1 | 1 | 1 | 1 | 1 | 1 | 1 |
| DishraS6 3 | 1 | 1 | 0 | 0 | 0 | 1 | 0 | 1 | 1 | 1 | 1 | 1 | 1 | 1 | 1 | 1 | 1 |
| Maliya jyoti-1 | 1 | 1 | 0 | 0 | 0 | 1 | 0 | 1 | 1 | 1 | 1 | 1 | 1 | 1 | 1 | 1 | 1 |
| Haripal 103 (1) | 1 | 1 | 0 | 0 | 0 | 1 | 0 | 1 | 1 | 1 | 1 | 1 | 1 | 1 | 1 | 1 | 1 |
| HaripalS6 1 | 1 | 1 | 0 | 0 | 0 | 1 | 0 | 1 | 1 | 1 | 1 | 1 | 1 | 1 | 1 | 1 | 1 |
| Maliya jyoti-2 | 1 | 1 | 0 | 0 | 0 | 1 | 0 | 1 | 1 | 1 | 1 | 1 | 1 | 1 | 1 | 1 | 1 |
| Samudragarh stem B | 1 | 1 | 0 | 0 | 0 | 1 | 0 | 1 | 1 | 1 | 1 | 1 | 1 | 1 | 1 | 1 | 1 |
| Modina joyti 1 | 1 | 1 | 0 | 0 | 0 | 1 | 0 | 1 | 1 | 1 | 1 | 1 | 1 | 1 | 1 | 1 | 1 |
| Dishra S6 4 | 1 | 1 | 0 | 0 | 0 | 1 | 0 | 1 | 1 | 1 | 1 | 1 | 1 | 1 | 1 | 1 | 1 |
| Sarisha jyoti 2 | 1 | 1 | 0 | 0 | 0 | 1 | 0 | 1 | 1 | 1 | 1 | 1 | 1 | 1 | 1 | 1 | 1 |
| Haripal 103 (2) | 1 | 1 | 0 | 0 | 0 | 1 | 0 | 1 | 1 | 1 | 1 | 1 | 1 | 1 | 1 | 1 | 1 |
| Polba sangrampur F2S6 | 1 | 1 | 0 | 0 | 0 | 1 | 0 | 1 | 1 | 1 | 1 | 1 | 1 | 1 | 1 | 1 | 1 |
| Mylliyam 1 | 1 | 1 | 0 | 0 | 0 | 1 | 0 | 1 | 1 | 1 | 1 | 1 | 1 | 1 | 1 | 1 | 1 |
| Mylliyam other side | 1 | 1 | 0 | 0 | 0 | 1 | 0 | 1 | 1 | 1 | 1 | 1 | 1 | 1 | 1 | 1 | 1 |
| Mawphlang jyoti | 1 | 1 | 0 | 0 | 0 | 1 | 0 | 1 | 1 | 1 | 1 | 1 | 1 | 1 | 1 | 1 | 1 |
| Mylliyam jyoti | 1 | 1 | 0 | 0 | 0 | 1 | 0 | 1 | 1 | 1 | 1 | 1 | 1 | 1 | 1 | 1 | 1 |
| Haldibari F1 (2) | 1 | 1 | 0 | 0 | 0 | 1 | 0 | 1 | 1 | 1 | 1 | 1 | 1 | 1 | 1 | 1 | 1 |
| Haripal 103 (3) | 1 | 1 | 0 | 0 | 0 | 1 | 0 | 1 | 1 | 1 | 1 | 1 | 1 | 1 | 1 | 1 | 1 |
| Samudragarh D | 1 | 1 | 0 | 0 | 0 | 1 | 0 | 1 | 1 | 1 | 1 | 1 | 1 | 1 | 1 | 1 | 1 |
| Sarisha Pukhraj 2 | 1 | 1 | 0 | 0 | 0 | 1 | 0 | 1 | 1 | 1 | 1 | 1 | 1 | 1 | 1 | 1 | 1 |
| Dinhata Tomato 1 | 1 | 1 | 0 | 0 | 0 | 1 | 0 | 1 | 1 | 1 | 1 | 1 | 1 | 1 | 1 | 1 | 1 |
| Dinhata Tomato 2 | 1 | 1 | 0 | 0 | 0 | 1 | 0 | 1 | 1 | 1 | 1 | 1 | 1 | 1 | 1 | 1 | 1 |
| Dinhata Tomato 3 | 1 | 1 | 0 | 0 | 0 | 1 | 0 | 1 | 1 | 1 | 1 | 1 | 1 | 1 | 1 | 1 | 1 |
| Barpeta Tomato 1 | 1 | 1 | 0 | 0 | 0 | 1 | 0 | 1 | 1 | 1 | 1 | 1 | 1 | 1 | 1 | 1 | 1 |
| Barpeta Tomato 2 | 1 | 1 | 0 | 0 | 0 | 1 | 0 | 1 | 1 | 1 | 1 | 1 | 1 | 1 | 1 | 1 | 1 |
| Barpeta Tomato 3 | 1 | 1 | 0 | 0 | 0 | 1 | 0 | 1 | 1 | 1 | 1 | 1 | 1 | 1 | 1 | 1 | 1 |
| Barpeta Tomato 4 | 1 | 1 | 0 | 0 | 0 | 1 | 0 | 1 | 1 | 1 | 1 | 1 | 1 | 1 | 1 | 1 | 1 |
| Barpeta Potato 2 | 1 | 1 | 0 | 0 | 0 | 1 | 0 | 1 | 1 | 1 | 1 | 1 | 1 | 1 | 1 | 1 | 1 |
| Panskura 1 | 1 | 1 | 0 | 0 | 0 | 1 | 0 | 1 | 1 | 1 | 1 | 1 | 1 | 1 | 1 | 1 | 1 |
| Panskura 2 | 1 | 1 | 0 | 0 | 0 | 1 | 0 | 1 | 1 | 1 | 1 | 1 | 1 | 1 | 1 | 1 | 1 |
| Panskura 3 | 1 | 1 | 0 | 0 | 0 | 1 | 0 | 1 | 1 | 1 | 1 | 1 | 1 | 1 | 1 | 1 | 1 |
| Panskura 4 | 1 | 1 | 0 | 0 | 0 | 1 | 0 | 1 | 1 | 1 | 1 | 1 | 1 | 1 | 1 | 1 | 1 |
| Panskura 5 | 1 | 1 | 0 | 0 | 0 | 1 | 0 | 1 | 1 | 1 | 1 | 1 | 1 | 1 | 1 | 1 | 1 |
| Sabour 2 | 1 | 1 | 0 | 0 | 0 | 1 | 0 | 1 | 1 | 1 | 1 | 1 | 1 | 1 | 1 | 1 | 1 |
| Sabour 3 | 1 | 1 | 0 | 0 | 0 | 1 | 0 | 1 | 1 | 1 | 1 | 1 | 1 | 1 | 1 | 1 | 1 |
| Sabour 4 | 1 | 1 | 0 | 0 | 0 | 1 | 0 | 1 | 1 | 1 | 1 | 1 | 1 | 1 | 1 | 1 | 1 |
| Sabour 6 | 1 | 1 | 0 | 0 | 0 | 1 | 0 | 1 | 1 | 1 | 1 | 1 | 1 | 1 | 1 | 1 | 1 |
| Sabour 7 | 1 | 1 | 0 | 0 | 0 | 1 | 0 | 1 | 1 | 1 | 1 | 1 | 1 | 1 | 1 | 1 | 1 |
| Sabour 8 | 1 | 1 | 0 | 0 | 0 | 1 | 0 | 1 | 1 | 1 | 1 | 1 | 1 | 1 | 1 | 1 | 1 |
| Sabour 9 | 1 | 1 | 0 | 0 | 0 | 1 | 0 | 1 | 1 | 1 | 1 | 1 | 1 | 1 | 1 | 1 | 1 |
| US-1 | 1 | 1 | 0 | 0 | 0 | 1 | 0 | 1 | 1 | 1 | 1 | 1 | 1 | 1 | 1 | 1 | 1 |
| 13_A2 (Blue_13) | 1 | 1 | 0 | 0 | 0 | 1 | 0 | 1 | 1 | 1 | 1 | 1 | 1 | 1 | 1 | 1 | 1 |

**Supplementary Table S2 online:** RG57 DNA fingerprinting profile of Eastern and North eastern Indian *Phytophthora infestans* population. Banding is indicated as follows: 0: No band observed; 1: Band observed.

|  |  |  |  | **Locus name** | | | | | | | | | | | |
| --- | --- | --- | --- | --- | --- | --- | --- | --- | --- | --- | --- | --- | --- | --- | --- |
|  |  |  |  | **Pi02** | **Pi4B** | **PiG11** | **Pi04** | **Pi63** | **Pi70** | **D13** | **PinfSSR11** | **PinfSSR2** | **PinfSSR4** | **PinfSSR6** | **PinfSSR8** |
| **Isolate Name** | **Host^a^** | **Region^b^** | **State** | **Allele size range255-275** | **Allele size range 200-295** | **Allele size range 130-206** | **Allele size range 160-175** | **Allele size range 265-280** | **Allele size range 185-205** | **Allele size range 100-210** | **Allele size range 325-360** | **Allele size range 165-180** | **Allele size range 280-305** | **Allele size range 230-250** | **Allele size range 250-275** |
| Chamurchi jyoti 2 | P | NEI | West Bengal | 266/268/0 | 203/205/213 | 154/158/160 | 166/170/0 | 273/279/0 | 192/192/0 | 136/138/156 | 341/341/0 | 173/173/0 | 284/292/0 | 240/244/0 | 260/266/0 |
| Dishra S6 4 | P | EI | West Bengal | 266/268/0 | 205/213/0 | 154/160/168 | 166/170/0 | 273/279/0 | 192/192/0 | 136/154/0 | 341/341/0 | 173/173/0 | 284/294/296 | 240/244/0 | 260/266/0 |
| Mawphlang jyoti | P | NEI | Meghalaya | 266/268/0 | 205/213/0 | 154/158/160 | 166/170/0 | 273/279/0 | 192/192/0 | 136/138/156 | 341/341/0 | 173/173/0 | 284/292/294 | 240/244/0 | 260/266/0 |
| Mylliyam jyoti | P | NEI | Meghalaya | 266/268/0 | 205/213/0 | 154/158/160 | 166/170/0 | 273/279/0 | 192/192/0 | 136/138/156 | 341/341/0 | 173/173/0 | 284/294/0 | 240/244/0 | 260/266/0 |
| Mylliyam other side | P | NEI | Meghalaya | 266/268/0 | 205/213/0 | 154/158/160 | 166/170/0 | 273/279/0 | 192/192/0 | 136/156/0 | 341/341/0 | 173/173/0 | 284/292/294 | 240/244/0 | 260/266/0 |
| Haldibari T 5 | T | NEI | West Bengal | 266/268/0 | 205/213/0 | 154/158/160 | 166/166/0 | 273/279/0 | 192/192/0 | 136/138/156 | 341/341/0 | 173/173/0 | 284/292/294 | 240/244/0 | 260/266/0 |
| Lataguri serpa alu 2 | P | NEI | West Bengal | 266/268/0 | 205/213/0 | 154/160/164 | 166/170/0 | 273/279/0 | 192/192/0 | 136/138/156 | 341/341/0 | 173/173/0 | 284/294/302 | 240/244/0 | 260/266/0 |
| Lataguri serpa alu 1 | P | NEI | West Bengal | 266/268/0 | 205/213/0 | 154/160/164 | 166/170/0 | 273/279/0 | 192/192/0 | 136/138/156 | 341/341/0 | 173/173/0 | 284/294/308 | 240/244/0 | 260/266/0 |
| Nadia chandirampur 1 | P | EI | West Bengal | 266/268/0 | 205/213/0 | 154/160/164 | 166/170/0 | 273/279/0 | 192/192/0 | 136/156/0 | 341/341/0 | 173/173/0 | 284/294/296 | 240/244/0 | 260/266/0 |
| Panskura 3 | P | EI | West bengal | 266/268/0 | 205/213/0 | 154/160/164 | 166/170/0 | 273/279/0 | 192/192/0 | 134/136/154 | 341/341/0 | 173/173/0 | 284/294/296 | 240/244/0 | 260/266/0 |
| Bonmukha jyoti | P | EI | West Bengal | 266/268/0 | 205/213/0 | 154/160/164 | 166/170/0 | 273/279/0 | 192/192/0 | 136/138/154 | 341/341/0 | 173/173/0 | 284/294/296 | 240/244/0 | 260/266/0 |
| Sarisha jyoti 2 | P | EI | West Bengal | 266/268/0 | 205/213/0 | 154/160/164 | 166/170/0 | 273/279/0 | 192/192/0 | 136/154/0 | 341/341/0 | 173/173/0 | 284/294/296 | 240/244/0 | 260/266/0 |
| Sabour 9 | T | EI | Bihar | 266/268/0 | 205/213/0 | 154/160/0 | 166/170/0 | 273/279/0 | 192/192/0 | 136/140/164 | 341/341/0 | 173/173/0 | 284/294/0 | 240/244/0 | 260/266/0 |
| Sabour 4 | T | EI | Bihar | 266/268/0 | 205/213/0 | 154/160/0 | 166/170/0 | 273/279/0 | 192/192/0 | 136/140/0 | 341/341/0 | 173/173/0 | 284/294/0 | 240/244/0 | 260/266/0 |
| Haldibari F-1 | T | NEI | West Bengal | 266/268/0 | 205/213/0 | 154/160/0 | 166/170/0 | 273/279/0 | 192/192/0 | 136/146/154 | 341/341/0 | 173/173/0 | 284/294/296 | 240/244/0 | 260/266/0 |
| HaldibariF1 (2) | T | NEI | West Bengal | 266/268/0 | 205/213/0 | 154/160/0 | 166/170/0 | 273/279/0 | 192/192/0 | 136/154/0 | 341/341/0 | 173/173/0 | 284/294/0 | 240/240/0 | 260/266/0 |
| Samudragarh stem B | P | EI | West Bengal | 266/268/0 | 205/205/0 | 154/160/164 | 166/170/0 | 273/279/0 | 192/192/0 | 136/138/154 | 341/341/0 | 173/173/0 | 284/294/296 | 240/244/0 | 260/266/0 |
| Sabour 2 | T | EI | Bihar | 266/268/0 | 205/205/0 | 154/160/0 | 166/170/0 | 273/279/0 | 192/192/0 | 136/140/164 | 341/341/0 | 173/173/0 | 284/294/0 | 240/244/0 | 260/260/0 |
| DishraS6 1 | P | EI | West Bengal | 266/266/0 | 205/213/0 | 154/160/164 | 166/170/0 | 273/279/0 | 192/192/0 | 136/154/0 | 341/341/0 | 173/173/0 | 284/294/296 | 240/244/0 | 260/266/0 |

**Supplementary Table S3 online:**Microsatellite data from 12-plex multilocus genotyping of nineteen new variants of 13_A2 genotype of *Phytophthora infestans* from eastern and northeastern India.

^a^ T= Tomato; P= PotatoEI= Eastern India; NEI= North East India

| **Population** | **Ia(p value)^a^** | 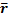***_d_* (p value)^b^** |
| --- | --- | --- |
| Eastern India | 0.997(0.001) | 0.238(0.001) |
| Eastern India – clone corrected | 0.281(0.083) | 0.060(0.002) |
| Northeastern India | 0.947(0.001) | 0.236(0.001) |
| Northeastern India – clone corrected | 0.275(0.094) | 0.064(0.005) |

**Supplementary Table S4 online: Linkage disequilibrium tests for populations of *Phytophthora infestans* in northeastern and eastern regions of India.** P values are based on 999 permutations.

**^a^**The index of association

**^b^**The standardized index of association

| 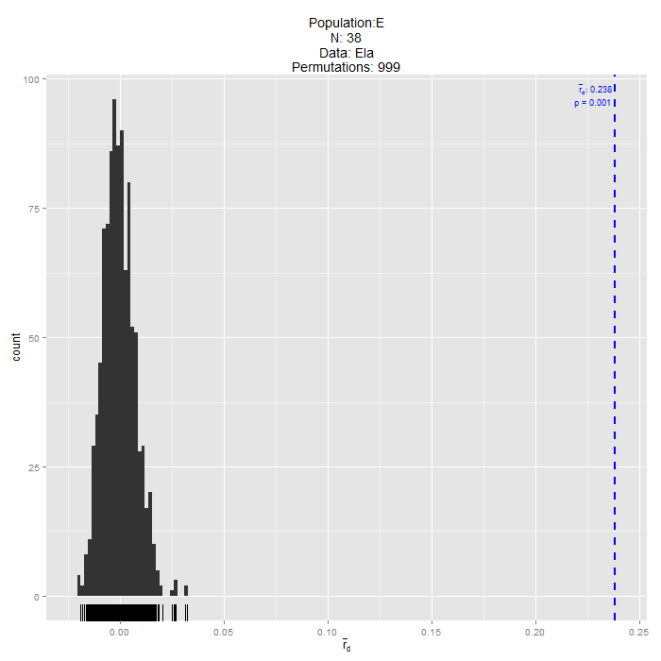 | 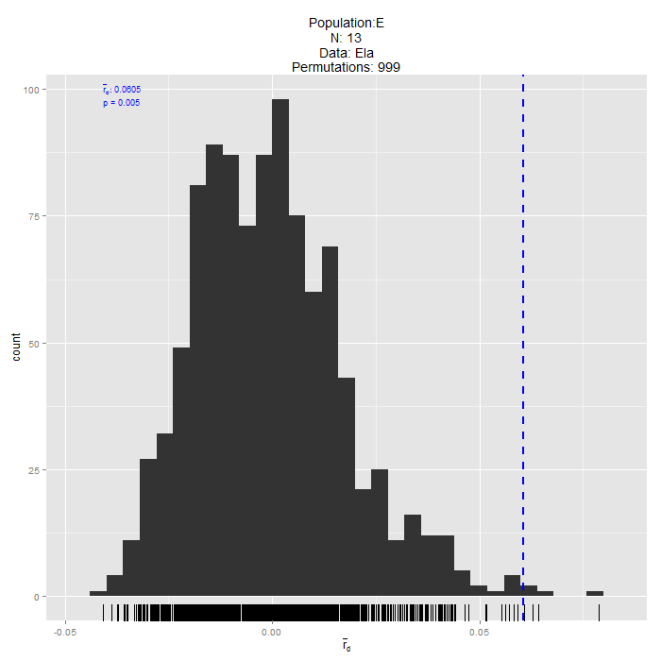 |
| --- | --- |
| 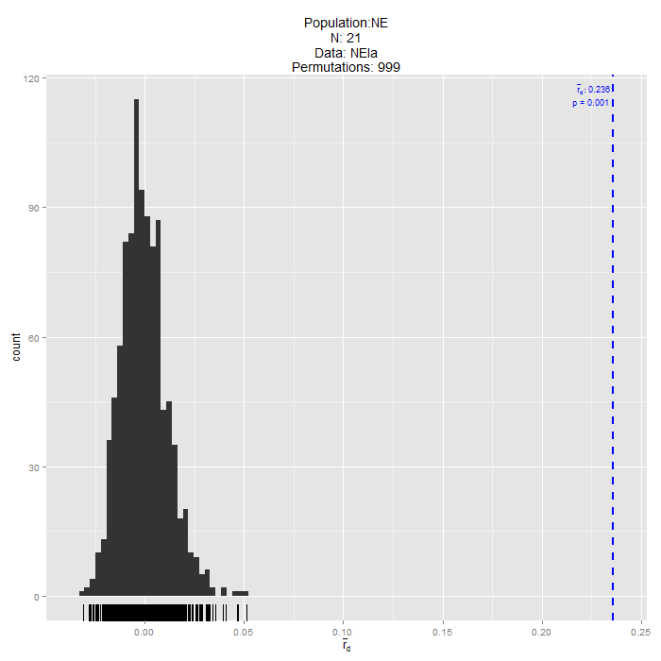 | 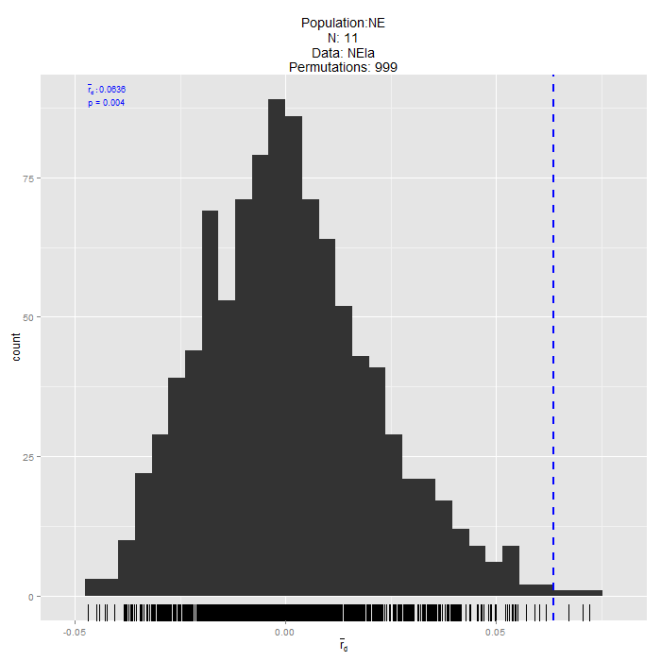 |

**Supplementary Fig. S5 online: Distribution of**
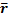
***_d_* (The standardized index of association) permutations for Eastern Indian (Population: E) and Northeastern Indian (Population: NE) populations of *Phytophthora infestans*.** Left side graphs utilize original data, while right side graphs utilize clone corrected data.

| **Population** | ***N***^a^ | **MLG**^b^ | **eMLG(SE)** ^c^ | **H**^d^ | **Hexp**^e^ | **Evenness** | **Ia**^f^ | 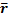***_d_***^g^ |
| --- | --- | --- | --- | --- | --- | --- | --- | --- |
| Potato | 67 | 18 | 12.7(1.56) | 2.10 | 0.425 | 0.512 | 1.137 | 0.359 |
| Tomato | 37 | 11 | 11(0) | 2.05 | 0.410 | 0.759 | 0.742 | 0.160 |
| All | 104 | 27 | 15.2(1.93) | 2.55 | 0.420 | 0.515 | 0.901 | 0.194 |

**Supplementary Table S6 online: Diversity statistics for all 12 loci in Indian populations of *Phytophthora infestans* based on host*.***

^a^*n*: number of individuals (not clone corrected);

^b^MLG: number of multilocus genotypes (MLG);

^c^eMLG: expected number of MLGs at smallest size of at least ten; SE: Standard error;

^d^H: Shannon-Weiner Index of MLG diversity;

^e^Hexp: Nei’s 1978 expected heterozygosity;

^f^Ia: Index of Association;

g
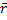
*_d_*: standardized index of association

| **MLG** | **Mean infection area (cm^2^) Potato cultivar Kufri pukhraj** | **Mean infection area (cm^2^) Potato cultivar Kufri jyoti** | **Mean infection area (cm^2^) Tomato cultivar Arka vikas** | **Host of origin** | **Geographic locations** | **Incubation period**  **Kufri pukhraj (Days)** | **Incubation period**  **Kufri jyoti (Days)** | **Incubation period**  **Arka vikas (Days)** | **Latent period**  **Kufri pukhraj (Days)** | **Latent period**  **Kufri jyoti (Days)** | **Latent period**  **Arka vikas (Days)** |
| --- | --- | --- | --- | --- | --- | --- | --- | --- | --- | --- | --- |
| 1 | 7.61 (0.044) | 3.34 (0.026) | 0.31 (0.032) | Potato | NEI | 3 | 3 | 3 | 5 | 5 | 5 |
| 2 | 1.12 (0.381) | 1.19 (0.478) | 0.46 (0.008) | Potato | EI | 3 | 3 | 3 | 5 | 5 | 5 |
| 3 | 5.81 (0.003) | 6.25 (0.304) | 0.43 (0.034) | Potato | EI | 3 | 3 | 3 | 5 | 5 | 5 |
| 4 | 5.76 (0.126) | 6.27 (0.540) | 1.82 (0.063) | Potato | NEI | 2 | 2 | 3 | 5 | 5 | 5 |
| 5 | 2.00 (0.434) | 2.25 (0.516) | 0.46 (0.085) | Potato | NEI | 2 | 2 | 3 | 5 | 5 | 5 |
| 6 | 3.59 (0.272) | 3.23(0.221) | 1.02 (0.113) | Potato | NEI | 2 | 2 | 3 | 5 | 5 | 5 |
| 7 | 5.86 (0.700) | 5.53 (0.670) | 0.75 (0.123) | Tomato | NEI | 3 | 3 | 3 | 5 | 5 | 5 |
| 8 | 0.91 (0.019) | 0.22 (0.006) | 0.19 (0.000) | Potato | NEI | 3 | 3 | 3 | 5 | 5 | Sporangia not seen |
| 9 | 0.93 (0.068) | 0.61 (0.142) | 0.38 (0.000) | Potato | NEI | 3 | 3 | 3 | 5 | 5 | 5 |
| 10 | 0.84 (0.184) | 0.78 (0.181) | 0.39 (0.052) | Potato | EI | 3 | 3 | 3 | 5 | 5 | 5 |
| 11 | 7.29 (1.196) | 7.07 (0.874) | 1.14 (0.312) | Potato | EI | 3 | 3 | 3 | 5 | 5 | 5 |
| 12 | 4.05 (0.384) | 3.18 (0.236) | 1.04 (0.082) | Potato | EI | 3 | 3 | 3 | 5 | 5 | 5 |
| 13 | 2.21 (0.363) | 3.0 (0.001) | 0.74 (0.139) | Potato | EI | 3 | 3 | 3 | 5 | 5 | 5 |
| 14 | 8.13 (0.663) | 6.51 (0.607) | 3.67 (0.114) | Potato | EI | 3 | 3 | 3 | 5 | 5 | 5 |
| 15 | 0.07 (0.025) | 0.08 (0.001) | 0.31 (0.056) | Tomato | EI | 5 | 3 | 3 | Sporangia not seen | Sporangia not seen | 5 |
| 16 | 2.76 (0.360) | 3.27 (0.810) | 3.04 (0.222) | Tomato | EI | 3 | 3 | 3 | 5 | 5 | 5 |
| 17 | 0.0 (0.0) | 0.0 (0.0) | 0.61 (0.056) | Tomato | NEI | - | - | 3 | - | - | 5 |
| 18 | 0.53 (0.094) | 0.87 (0.007) | 0.56 (0.226) | Potato | EI | 3 | 3 | 3 | 5 | 5 | 5 |
| 19 | 3.05 (1.130) | 3.72 (1.067) | 1.26 (0.512) | Tomato | NEI | 3 | 3 | 3 | 5 | 5 | 5 |
| 20 | 2.06 (0.628) | 2.77 (0.076) | 2.76 (0.005) | Potato | NEI | 3 | 3 | 3 | 5 | 5 | 5 |
| 20 | 2.09 (0.567) | 2.44 (0.405) | 2.89 (0.116) | Tomato | NEI | 3 | 3 | 3 | 5 | 5 | 5 |
| 21 | 0.0 (0.0) | 0.0 (0.0) | 0.58 (0.049) | Tomato | NEI | - | - | 3 | - | - | 5 |
| 22 | 0.0 (0.0) | 0.21 (0.044) | 0.0 (0.0) | Potato | EI | - | 3 | - | - | Sporangia not seen | - |
| 23 | 0.48(0.181) | 0.51( 0.103) | 0.76 (0.121) | Tomato | EI | 3 | 3 | 3 | 5 | 5 | 5 |
| 24 | 3.28 (0.003) | 4.93 (1.766) | 0.49 (0.113) | Potato | EI | 3 | 3 | 3 | 5 | 5 | 5 |

**Supplementary Table S8 online : MLG= Multilocus genotype,** Mean infection area, incubation and latent period of 24 MLGs of *Phytophthora infestans* on two potato ( ‘Kufri jyoti’, Kufri pukhraj’)and a tomato (‘Arka vikas’) cultivar.

Standard error values are on bracket.


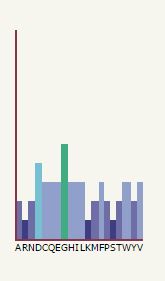


**Supplementary Fig. S10 online: Phyre2 investigator predict that the mutation in Phytophthora infestans with highest likelihood to affect function at position 124 is glycine**

The mutational analysis graph represents the predicted effect of mutations at position 124 in AVR3a protein sequence. These predictions are made using the SuSPect method.The 20 possible amino acid types are labelled along the x-axis with their one-letter code. The coloured bars indicating the probability that a mutation to the corresponding residue will have some effect on function of the protein or on the phenotype of the organism. Short and blue bars indicate unlikely mutation effects on functions while tall and other colour bars indicate mutations likely to affect functions. In position 124,the mutation with the highest likelihood comes from arginine to glycine.
